# App-based supplemental exercise during inpatient orthopaedic rehabilitation increases activity levels: a pilot randomised control trial

**DOI:** 10.1186/s40814-019-0430-9

**Published:** 2019-03-16

**Authors:** Tram Bui, Clayton King, Ana Llado, Darren Lee, Grace Leong, Anuka Paraparum, Ingrid Li, Katharine Scrivener

**Affiliations:** 1 Royal Rehab, 235 Morrison Road, Ryde, NSW 2112 Australia; 2 MQ Health Physiotherapy, Suite 307, Level 3, 2 Technology Place, Macquarie Park, NSW 2109 Australia; 3 Department of Health Professions, Macquarie University, Ground Floor, 75 Talavera Road, Macquarie Park, NSW 2113 Australia

**Keywords:** Orthopaedic rehabilitation, Exercise therapy, Outcomes, Physical therapy, mHealth

## Abstract

**Background:**

There is a known positive relationship between time in therapy and therapy outcomes. Effective rehabilitation should therefore include larger doses of therapy. However, individuals participating in inpatient rehabilitation have low levels of activity throughout the day. This level of inactivity may limit rehabilitation potential. New technologies which deliver personalised exercise programs and track time spent on exercises may lead to greater activity levels and therefore improve functional outcomes in rehabilitation. This pilot randomised control trial aimed to investigate whether an app-based supplemental exercise program in orthopaedic rehabilitation will be feasible and acceptable to participants, increase activity levels and improve functional outcomes.

**Methods:**

Participants were randomised to receive supplemental exercise via an app (PTPal™) on a tablet device additional to usual care or usual care alone. Primary outcome measures were participant satisfaction with app-based supplemental exercise, total repetitions of each activity and time in supplemental exercise programs. Secondary measures were 10-m walk test (10MWT), 6-min walk test (6MWT), Timed Up and Go (TUG), Functional Independence Measure and length of stay assessed by a blinded assessor.

**Results:**

Twenty individuals admitted into an inpatient private general rehabilitation unit for orthopaedic rehabilitation over a 4-week duration were included in this study. High acceptance of the app-based supplemental exercise program was demonstrated. Those using the app completed an additional 549 exercise repetitions during their admission (694 supplemental app-based repetitions vs 146 supplemental paper-based repetitions in the control group, mean difference [MD] 549, 95% CI 95 to 1002, *p* = 0.02) and an additional 157 min in supplemental exercise throughout their admission (195.3 min vs 38.7 min, MD 157 min, 95% CI 0.9–312.3 min, *p* = 0.05). There was insufficient power to demonstrate statistical significance in functional outcomes, but a trend towards improved functional outcomes was observed in the intervention group.

**Conclusion:**

An app-based exercise program increases activity levels, is feasible and is a safe intervention with the potential to improve functional outcomes. This pilot study should be followed with a larger study powered to demonstrate functional effects with more participants having greater impairment.

**Trial registration:**

Australian New Zealand Clinical Trials Registry (ANZCTR); trial number ACTRN12617000817347. This study was retrospectively registered (registration date 05/06/2017).

## Background

New technologies which deliver personalised exercise programs and track time spent on the exercises may lead to greater activity levels and therefore improve functional outcomes in rehabilitation [1]. Such technology is already available to the layperson, however has not been utilised in hospitals to improve delivery of health programs, despite their acceptability as a health promotion aid in the community [2]. There is research to demonstrate that in the community, use of a Fitbit for 6 months did not result in any improvements in health outcomes [3]. However, people have reported an independent or supplementary exercise program, prescribed when they are in hospital, to be enjoyable and beneficial [4]. In this inpatient hospital setting, where people are motivated to improve and are receiving close supervision, it is possible that supplementing scheduled therapy with a supplemental exercise program may be effective and may result in benefits not seen in the community.

From a hospital viewpoint, using a standardised exercise program via an app may help to standardise therapy hours and improve access to rehabilitation programs, providing more efficiencies in service. It is known that variations exist between different institutions in terms of therapy hours offered, delivery and measurement of rehabilitation programs. A study of institutional variation in brain injury rehabilitation demonstrated that the difference between those programs which delivered the most and those that delivered the least found that “the centers with the highest mean total weekday hours of occupational, physical, and speech therapies delivered twice as much therapy as the lowest center” [5]. In addition, there were differences between the types of therapy offered and diversity of therapists’ experience. Variations may exist due to a lack of resources, with rehabilitation acknowledged as “an expensive and limited resource” [6]. These resource limitations are likely to impact on outcomes.

There is a known positive relationship between time in therapy and therapy outcomes, with larger doses of therapy leading to clinically meaningful improvements [7]; however, due to institutional limitations and person specific factors, inpatients do not always complete optimal amounts of physical activity. Effective rehabilitation should include high levels of physical activity [8]; however, most individuals residing in rehabilitation facilities spend a substantial amount of time alone, inactive or sleeping [9]. Published research demonstrates that individuals participating in inpatient rehabilitation have low levels of activity and complete less activity than recommended for healthy older adults, with a total of 8 min of walking and 398 steps per day [10, 11]. Individuals in rehabilitation often spend large proportions of their day completing non-therapeutic activities [12, 13]. This level of inactivity may limit rehabilitation potential.

Physical activity is “any bodily movement produced by skeletal muscles that requires energy expenditure” [14] and includes both planned/structured activity and incidental activity. One strategy to increase activity levels in rehabilitation is to increase the amount of exercise individuals can undertake in their own time, outside of scheduled therapy hours [15]. There is evidence that supplemental programs can improve functional outcomes; however, research demonstrates that adherence to independent exercise programs is generally low [16].

The research questions were:

1. Is an app-based exercise program, delivered via a tablet device, acceptable and feasible for inpatients receiving orthopaedic rehabilitation?
2. Will an app-based exercise program increase activity levels of individuals participating in inpatient rehabilitation during their out-of-therapy hours, specifically in relation to the amount of exercise completed?
3. Will an app-based exercise program increase physical and functional outcomes of participants?

## Method

### Design

Northern Sydney Local Health District, Human Research Ethics Committee approved the study (HREC reference: HREC/14/HAWKE/444). Site-specific ethics was obtained (17SSA02). All participants provided written informed consent before data collection commenced.

Individuals admitted for orthopaedic rehabilitation were randomised to either usual care orthopaedic program or to usual care with an app-based supplemental exercise program, delivered via an app downloaded to a tablet device (PTPal™). This single-center, single-blind randomised control trial was conducted at a private rehabilitation facility in the Sydney metropolitan area over 4 weeks. Twenty participants were recruited and allocated randomly to either group. Twenty was chosen as a robust number to determine feasibility of the program. The primary outcome was use and acceptability to participants. Use was measured as the amount of time on and number of repetitions completed using the app. Acceptability was measured by conducting a survey at discharge. In addition, physical measures were conducted—these included the 10-m walk test (10MWT), 6-min walk test (6MWT) and Timed Up and Go (TUG). Total length of stay (LOS) in inpatient unit and Functional Independence Measure (FIM) were also assessed.

### Participants

Recruitment of 20 participants was planned—this was selected as a robust number to determine feasibility. Participants were included if they were inpatients undergoing usual care orthopaedic rehabilitation, aged over 18 years of age, able to consent, had been admitted with an orthopaedic diagnosis, were willing to use or be educated on the use of the tablet device and had no medical contraindications to a supplemental exercise program. If the individual was unable to provide consent due to cognitive impairment defined as a Mini Mental State Examination (MMSE) score less than 24/30, they were not approached to take part in the study.

### Intervention

Participants were each randomised according to a number drawn from a concealed box and allocated by one of the researchers to the intervention or control groups. The intervention group received a supplementary exercise program designed by their treating therapist and uploaded to a tablet device in addition to usual care. A tablet device was provided to the intervention group for the duration of their inpatient program. The control group received usual care, which may have included encouragement to undertake supplemental exercise, either with instructions on paper or verbally according to the therapist. All control participants received a paper diary to record the amount and number of repetitions of supplemental exercise (if they were recommended)—this was collected by the research assistant on discharge.

Exercise programs for the intervention participants were individually designed by the treating therapist. PTPal™ is a care delivery app that allows clinicians and therapists to send individuals digital prescriptions of exercises directly to a mobile or tablet device. For this project, participants had a de-identified login created by the research team and an individualised exercise program was uploaded to a Royal Rehab Apple iPad Air 2™ that was provided to the participants for the duration of their admission. Exercise programs included the following exercise types: range of motion, stretching, strengthening and practice of everyday tasks, e.g. walking or standing up. The time that participants logged onto their exercise account, repetitions undertaken and difficulties encountered were remotely monitored by the treating therapist and principal investigators. After allocation to either group, the treating therapist was made aware of their allocation, in order to allow them to design the supplemental exercise program. The intervention participants received one session at the commencement of their program to learn to use the app, and ongoing support to both groups was provided, as needed, to the participants by the research assistant.

### Outcome measures

1. *Primary outcome:* quantitative data from app from intervention participants and diaries from controls regarding the amount of time and number of repetitions in supplemental exercise. Qualitative data obtained from a survey administered at the completion of inpatient rehabilitation to assess satisfaction with the app.
2. *Secondary outcome:* physical outcomes of participants undergoing orthopaedic inpatient rehabilitation, measured by 6MWT, 10MWT and Time Up and Go. Additional measures obtained were total length of stay and total hours of inpatient therapy as measured by their inpatient timetable.

Demographic measures including length of stay and total hours of scheduled therapy were collected upon discharge by the principal investigator to determine their diagnosis, co-morbidities, medications and length of stay.

Data regarding supplemental exercise for the intervention group were reported from the app which recorded information regarding total time, type of exercise, number of repetitions, sets and perceived difficulty as measured by participants. The app automatically counts the number of reps and computes total time when an exercise is selected; thus, the data are accurate if the participants are engaged with the program. This de-identified information was provided to the researchers by PTPal™ at the completion of the project, after all participants had been discharged.

Data regarding supplemental exercise for the control group were collected from their paper diaries, which was collected by the research assistant on discharge from hospital. Participants recorded the exercise they completed in a paper form provided. This form of quantifying exercise dose has been shown to be valid in a rehabilitation setting [17].

A paper questionnaire was given to the intervention group to determine their satisfaction with the app-based exercise program.

To compare the amount of usual care therapy between groups, the total hours of inpatient therapy was measured by their inpatient timetable and summated by one of the research assistants after discharge.

A blinded assessor conducted physical measures at baseline and discharge, including 10MWT, 6MWT, TUG and FIM. The blinded assessor was a physiotherapist who is a faculty member with Macquarie University. Blinding was important in order to remove any possibility of actual or perceived bias.

The 6MWT is a self-paced test which assesses distance walked over 6 min as a sub-maximal test of aerobic capacity [18]. This test is often performed before and after intervention to assess for a clinically significant improvement and has shown excellent short-term reproducibility [19].

The 10MWT assesses walking speed in metres per second over a distance of 10 m. It is a safe test that can be easily implemented with minimal facilities and budget [20].

The TUG test is a determinant of falls risk and is used to measure the progress of balance, sit to stand and walking. Originally designed for the elderly population, it is now used in a variety of settings. The test involves the participant standing from a seated position, walking 3 m, turning around and returning to sitting in the chair. Total test time of 14 s or longer has been related to a high risk for falling [21].

The FIM is a universally recognised indicator of severity of disability and is used to assess improvement during a rehabilitation episode. This test is comprised of 18 items (13 motor tasks and 5 cognitive tasks) which are assessed on a 7-point ordinal scale. Total score indicates level of function and can range from 18 (total assistance) to 126 (complete independence). The test is used to measure functional change during a rehabilitation episode and is generally administered at admission and discharge [22].

### Data analysis

The demographic characteristics of all participants were described. A qualitative analysis of the intervention group survey results was undertaken and described. For measures of exercise dosage, independent *t* tests were used to compare the means between groups. For all measures of physical performance independent *t* tests were used to compare the mean change scores between the two groups. Statistical significance for all tests was set at *p* < 0.05.

## Results

### Participants

**Fig. 1:**
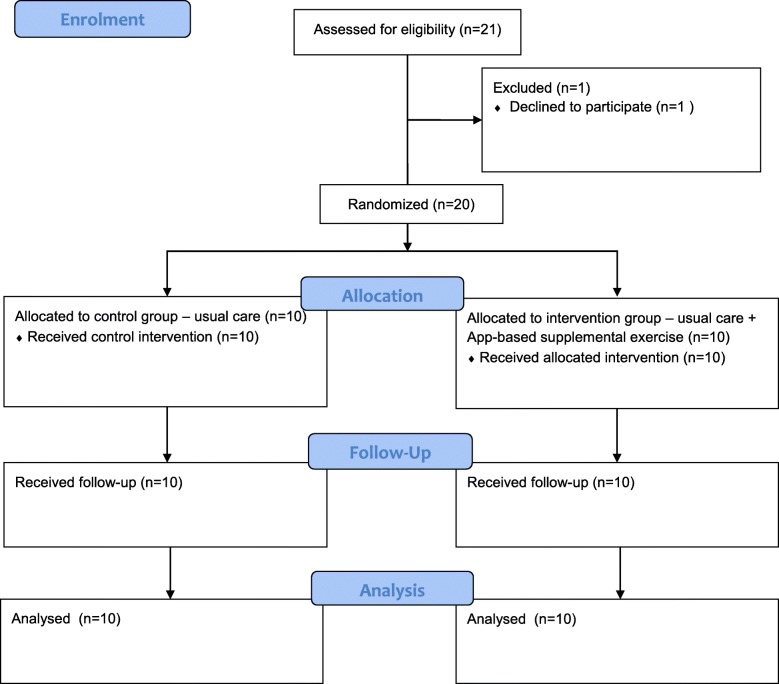
Participant flow diagram

All eligible individuals admitted to rehabilitation during this 4-week period were identified and invited to participate in the research project. Only one individual declined to participate due to concerns regarding their ability to complete supplemental exercise in addition to usual therapy. Twenty participants were recruited into the study. The flow of participants through the study is presented in Fig. 1.

Demographic information and admission measures for the participants can be found in Table 1. The intervention and control groups were similar for all measures.

**Table 1:** Admission (baseline) characteristics of the intervention vs control group

	Intervention (*n* = 10) (SD)	Control (*n* = 10) (SD)
Age (years)	65 (20.3)	66.3 (11.8)
Diagnosis	10 (3 hip, 5 knee, 2 other)	10 (2 hip, 7 knee, 1 other)
Gender (female)	7	5
MMSE (mean)	29 (1.9)	29.1 (0.7)
Average time/day in therapy (min)	96.4 (23.1)	95.6 (24.1)
FIM	105.9 (7.9)	106.9 (6.0)
6MWT (m)	207.9 (101.3)	196.9 (*N* = 9)* (61.7)
10MWT (s)	23.2 (30.0)	17.3 (8.1)
TUG (s)	26.5 (35.4)	23.1 (*N* = 9)* (8.4)
LOS (days)	12 (3.5)	10 (3.1)

*N = 9 as one participant was unable to perform the test

### Acceptability and feasibility of the intervention

The survey results demonstrated high acceptance by intervention participants in the use of technology to deliver a supplemental exercise program, even for those older participants who had previously not utilised tablet devices for information. Nine out of ten participants reported no need for extra help to access or use the tablet device. The participants were positive about the effects of supplemental exercise stating “it provided motivation to do extra exercise”, “useful and something I can do at home”, “it kept me focused”, “improved my flexibility” and “it helped me recover quickly”. There were no adverse effects related to supplementary exercise in either intervention or control group.

### Amount of supplementary exercise completed

The majority of intervention participants undertook additional exercise. The total amount of supplemental exercise varied from 5.9 min (for a participant who stated that they did more than this amount of exercise but due to no Internet connection time on the app was not logged) to 618 min.

### Effect of intervention on activity levels in supplemental exercise

In the intervention group, all of the participants completed a supplementary exercise program. The average amount of additional exercise in this group was 195 min during the admission. There was large variability in both additional time with SD 214 min, range 5.9 to 618 and repetitions with SD 590, range 55 to 1945.

**Fig. 2:**
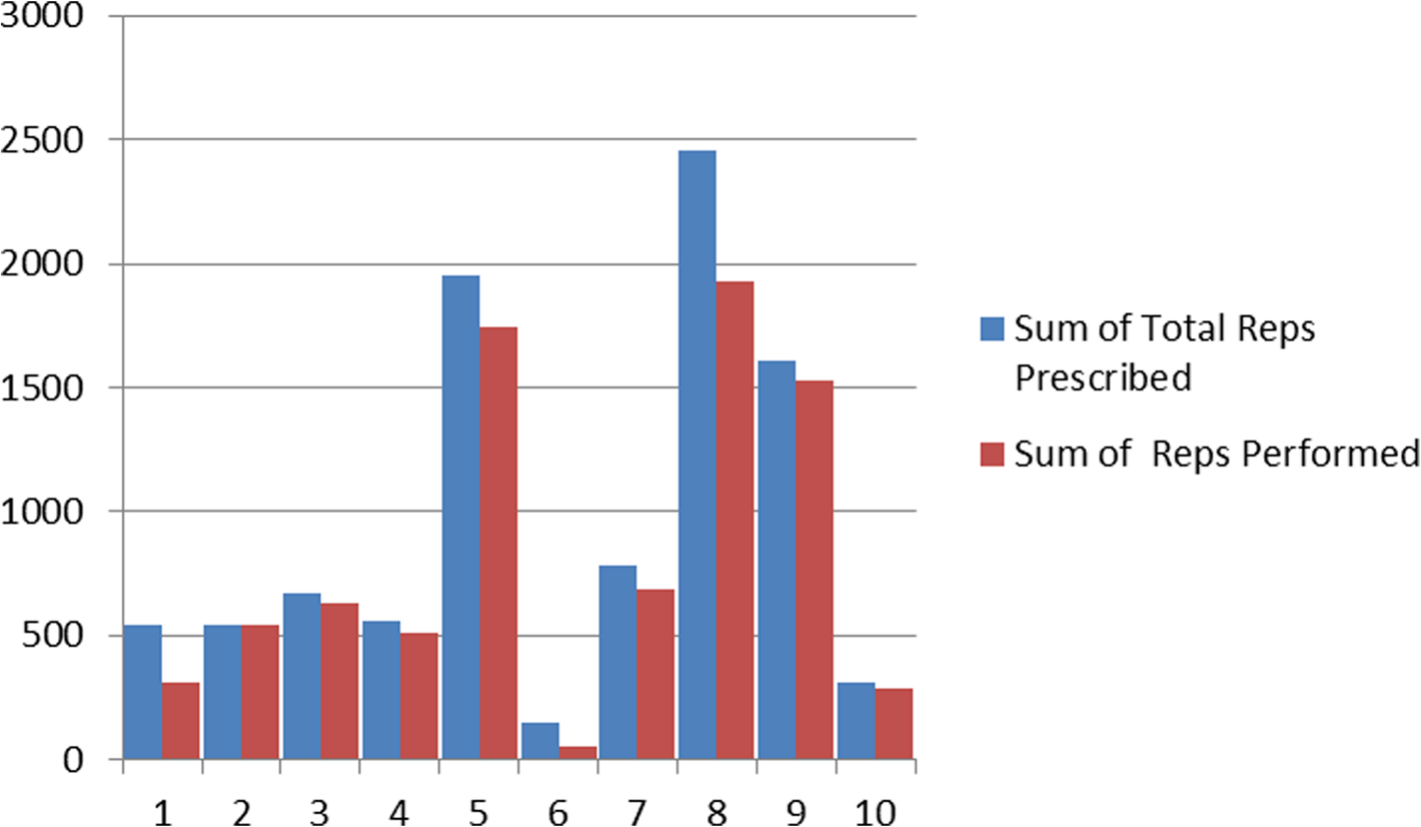
Repetitions prescribed vs repetitions performed

Most participants demonstrated good adherence, completing most of the prescribed exercise program. Figure 2 illustrates the number of exercise repetitions prescribed by the therapist compared to completed by the participant. Of the exercises attempted, participants completed 82.7% of the repetitions that were prescribed.

In the control group, three of the ten participants completed a supplementary exercise program. The average amount of additional exercise participants in this group reported completing was 39 min during the admission. There was large variability in both the additional time (SD 96 min, range 0 to 307) and the repetitions that were reported (SD 343, range 0 to 1053).

There was also an increase in supplemental exercise time between the app-based supplemental exercise group and the control group (refer to Table 2 for further details). This demonstrates a statistically significant difference between the number of repetitions between the groups (*p* = 0.02), with the intervention group having an average of 549 reps more in total compared with the control group.

**Table 2:** Activity levels in supplemental exercise, *n* = 20

	Intervention mean (SD)	Control mean (SD)	Between groups mean difference (95% CI)
Repetitions	694.2 (590.2)	145.5 (342.7)	548.7 (95.3 to 1002.1) *p* = 0.020
Time (min)	195.3 (213.8)	38.7 (96.1)	156.6 (0.9 to 312.3) *p* = 0.049

### Effect of intervention on functional outcomes

There was no statistically significant difference between the two groups in functional outcomes, as measured by FIM change, 6MWT, 10MWT, timed up and go and length of stay. Details of these results can be seen in Table 3.

**Table 3:** Functional outcomes, *n* = 20

	Intervention mean (SD)	Control mean (SD)	Between groups mean difference (95% CI)
LOS (days)	12.0 (3.5)	10.8 (3.0)	1.2 (− 1.8 to 4.2) *p* = 0.42
Change in FIM	14.9 (5.5)	15.5 (5.2)	− 0.6 (− 5.7 to 4.5) *p* = 0.81
FIM change/LOS	1.3 (0.5)	1.56 (0.6)	0.3 (− 0.8 to 0.3) *p* = 0.33
Change in 6MWT (m)	129.6 (63.0)	120.6 (*N* = 9)* (43.5)	9.0 (− 44.0 to 62.1) *p* = 0.72
6MWT/LOS (m/day)	11.3 (5.4)	10.9 (*N* = 9)* (7.5)	0.4 (− 5.8 to 6.5) *p* = 0.90
Change in 10MWT (m)	− 13.9 (28.2)	− 7.6 (7.6)	− 6.3 (− 25.8 to 13.1) *p* = 0.50
10MWT/LOS (m/day)	− 0.91 (1.4)	− 0.83 (1.0)	− 0.08 (− 1.2 to 1.0) p = 0.90
Change in TUG (s)	− 14.3 (29.2)	− 11.5 (7.5)	− 2.8 (− 24.0 to 18.4) *p* = 0.78
TUG/LOS (s/day)	− 0.95 (1.4)	1.04 (0.9)	0.09 (− 1.1 to 1.2) *p* = 0.87

*N = 9 due to missing data

## Discussion

This novel study has demonstrated the feasibility of using an app-based supplemental exercise program to increase activity levels in an inpatient rehabilitation setting. Despite its promise, this result is derived from a pilot study of 20 participants and will require a larger, adequately powered study in order to reach a definitive conclusion as to its clinical utility. The supplemental exercise program we examined was safe, without any adverse events, and supports previous research that individuals in rehabilitation enjoy supplementary exercise programs to augment formal therapy sessions [16] although this is the first study that has utilised an exercise app to demonstrate this in an inpatient setting. These results would be generalisable to other inpatient orthopaedic rehabilitation units.

This acceptance of an app-based exercise program in an inpatient setting has important implications for increasing the scope of physiotherapy practice to remotely monitor and upgrade activity in conjunction with scheduled face-to-face therapy, while being able to assess an individual’s comfort with the exercise program, providing encouragement as needed. Compliance to exercise programs is variable, with rates ranging from 30 to 70% published in current literature, and the majority of studies being completed in an outpatient setting [23–25, 26, 27]. In comparison, our compliance rate of 82.7% is high and exceeds 70%, which is the cut-off value often used to indicate sufficient adherence [16]. Within the rehabilitation setting, app-based exercise programs have the potential to be utilised after hours, especially on weekends, where there is known to be a reduction in physiotherapy access. Increasing physiotherapist hours will come at a greater cost after-hours and this study demonstrates that individuals are compliant and able to participate in a remotely delivered exercise program. There is also the potential for this program to deliver other therapy programs such occupational therapy and speech pathology.

However, this study demonstrated a wide variation in the amount of time that participants spent on the app and variety of exercises that were prescribed. There is the potential that with a higher quality of graphics, personalised content in the app improved therapists’ familiarity with the app, as well as improved institutional internet access, there may be greater uptake and participation by both therapists and participants. Use of an app to augment existing inpatient therapy programs has implications in resource allocation for both inpatient and outpatient settings. Using an app-based program has the potential to standardise evidence-based therapies, reducing the wide variation in practices that exists between facilities.

This study also demonstrated high recruitment potential, with the majority of individuals consenting (95%), within a 4-week window. These results suggest that recruitment for a larger trial would be achieved expeditiously although expanding our inclusion criteria to include more diverse diagnoses may result in a greater exclusion rate due to a higher prevalence of cognitive impairment in those with a neurological diagnosis.

This study demonstrates a trend towards functional improvements which may be more apparent in a larger study with participants with greater impairment, more diverse diagnoses especially in those diagnostic groups which require longer lengths of inpatient rehabilitation and should be followed with a larger study to evaluate its effects.

### Limitations

This study had limitations as it was a pilot study and not powered to detect significant changes in mobility. The functional significance of the supplementary exercise program was diluted by the significant amount of time that participants in both groups spent in scheduled therapy, on average 100 min each. The participants were on average high functioning with shorter lengths of stay, which limited the improvements they were likely to make. There was no follow-up time to evaluate the longer-term impact of increasing independent exercise on participants’ function, confidence and activity levels after discharge. Future studies may also examine the cost effectiveness of a supplemental app-based exercise program to allow clinicians to improve resource efficiency.

## Conclusion

An app-based exercise program is an acceptable and feasible method of increasing activity levels in orthopaedic rehabilitation. As a safe intervention, it also demonstrates the potential to improve functional outcomes. This pilot study should be followed with a larger study with more diverse diagnoses and greater impairments to determine its effectiveness.

## Abbreviations

10MWT: 10-m walk test
6MWT: 6-min walk test
FIM: Functional Independence Measure
LOS: Length of stay
MMSE: Mini Mental State Exam
TUG: Timed Up and Go
